# Synthesis and Analysis of Resorcinol-Acetone Copolymer

**DOI:** 10.3390/molecules14010364

**Published:** 2009-01-13

**Authors:** Ataru Kobayashi, Gen-ichi Konishi

**Affiliations:** Department of Organic & Polymeric Materials, Graduate School of Science & Engineering, Tokyo Institute of Technology, SORST, Japan

**Keywords:** Resorcinol, Acetone, Phenolic resin, Chromane ring, Addition-condensation, Cyclization.

## Abstract

Synthesis and characterization of resorcinol-acetone copolymer is described. The polymer was prepared by trifluoroacetic acid-catalyzed polymerization of resorcinol with acetone. According to the ^1^H-NMR, ^13^C-NMR, and MALDI-TOF Mass spectra data, the obtained polymer had three types of repeating units: isopropylidene bridged-resorcinol, chromane ring, and spiro-shaped double chromane ring, indicating that polymerization proceeded *via* simultaneous addition-condensation and cyclization of resorcinol with acetone. The obtained polymer can be useful not only for the development of plastic materials such as thermosets, adhesives, and coatings but also for the synthesis of biomaterials such as antimicrobial agents, pesticides, and medicines.

## Introduction

Resorcinol is the 1,3-isomer of dihydroxyphenol and is used as a chemical intermediate for the synthesis of pharmaceuticals, functional organic compounds, and polymer materials. In particular, phenolic resins obtained from resorcinols have been extensively investigated. Resorcinol novolac (**RN**) [1] and calixresorcinarenes (**CRs**) [2,3] are the two well-known resins derived from resorcinol. **RN** is a resorcinol-formaldehyde copolymer prepared using an acidic catalyst. It has many phenolic hydroxy groups and an aromatic ring, both of which are highly reactive. Consequently, **RN** has been extensively investigated for use in thermosets, adhesives, and as precursors for carbon materials [4]. On the other hand, **CRs** are cyclic oligomers derived from the hydroalkylation product of resorcinol and various alkyl or aryl aldehydes. **CRs** have hydrophobic cavities that can accommodate small molecules or ions and therefore, **CRs** have been extensively investigated in host-guest chemistry and analytical science [5,6]. 

**Figure 1:**
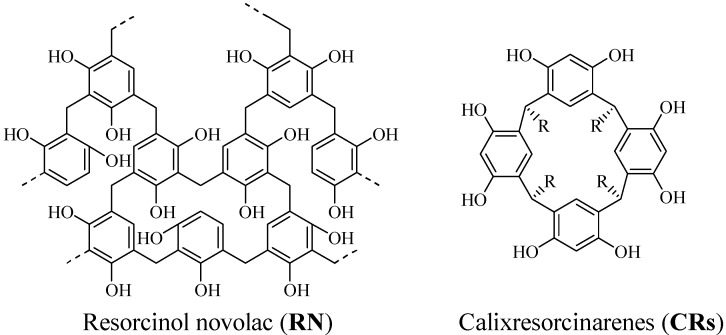
Resorcinol novolac and calix [4] resorcinarenes.

It would be useful to extend the applicability of **RN** in the field of material sciences by using other carbonyl compounds such as acetone. However, to the best of our knowledge, a well-defined resorcinol-acetone novolac has not yet been synthesized. While performing phenol-formaldehyde condensation, it has been observed that the reactivity of acetone is lower than that of the aldehyde because of steric hindrance. However, the use of phenol-acetone as a starting material, facilitates the production of bisphenol A. The novolac obtained from acetone involves the isopropylidene bridge in the polymer backbone, therefore, it should be more processable than a formaldehyde-based novolac [7,8,9]. In this paper, we report the acid-catalyzed polymerization of resorcinol derivatives with acetone and the characterization of the obtained polymers. These polymers are interesting from the viewpoint of both polymer materials and bio-related materials. 

## Results and Discussion

### Synthesis

**Scheme 1:**
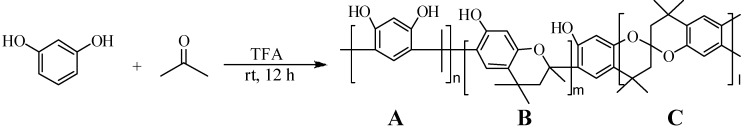
Synthesis of phenolic polymer **1**.

To prepare resorcinol-acetone copolymer, we determined appropriate polymerization conditions such as catalyst, solvent, and reaction temperature. The successful procedure for the preparation of resorcinol-acetone copolymer is as follows (Scheme 1): trifluoroacetic acid was added dropwise to a solution of resorcinol and excess acetone at 0 °C. After stirring the resulting solution for 12 h at room temperature, it was poured into water and the precipitate was collected. Further purification was carried out by reprecipitation in water. Polymer **1** was obtained as a colorless precipitate. The obtained polymer was readily soluble in common organic solvents such as tetrahydrofuran (THF), chloroform, and acetone. The result of the gel-permeation chromatography (GPC) analysis (eluent: THF; polystyrene standards) showed that the weight-average molecular weight (*M*_w_) of **1** was 3,700 (polydispersity index: *M*_w_/*M*_n_ = 2.3). The molecular weight and yield of this polymer were not dependent of the reaction temperature and polymerization time. For example, in the case of a higher reaction temperature (60 °C) (Condition A) or a longer polymerization time (24 h) (Condition B), similar polymers (Condition A: *M*_w_ = 2,600, *M*_w_/*M*_n_ = 1.8.; Condition B: *M*_w_ = 1,000, *M*_w_/*M*_n_ = 1.3.) were obtained.

**Figure 2:**
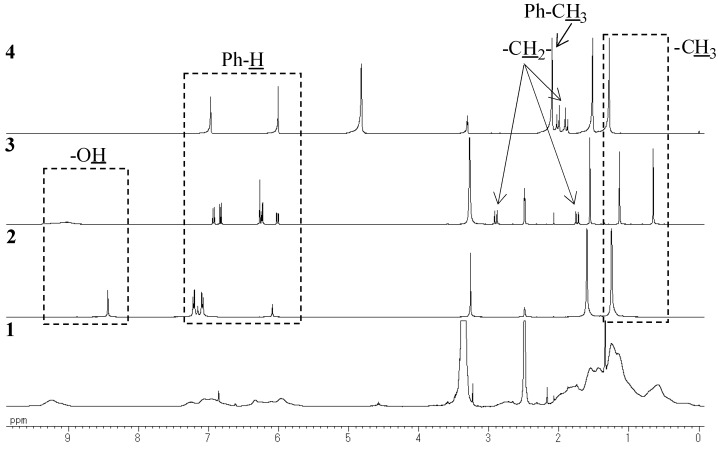
^1^H-NMR spectra of **1**, **2**, **3** (DMSO, 400 MHz), and **4** (MeOH, 400 MHz).

**Figure 3:**
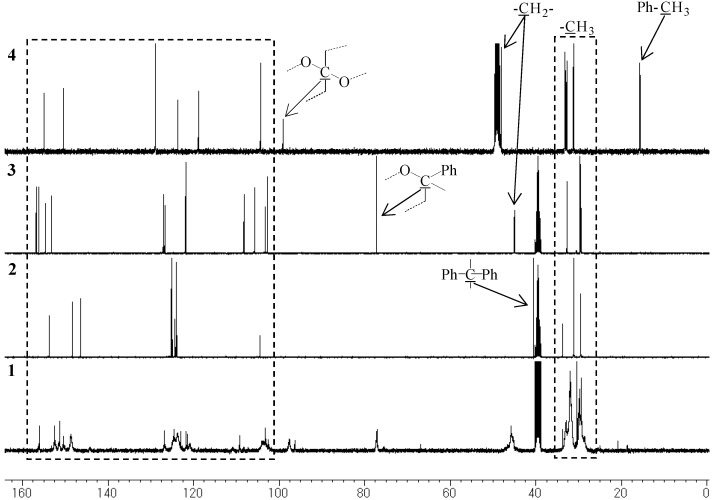
^13^C-NMR spectra of **1**, **2**, **3** (DMSO, 100 MHz), and **4** (MeOH, 100 MHz).

**Figure 4:**
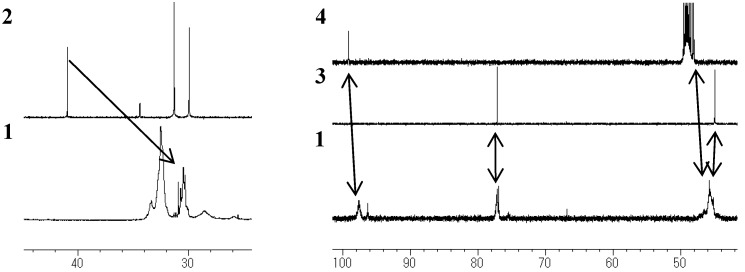
^13^C-NMR spectra of **1**, **2**, **3** (DMSO, 100 MHz), and **4** (MeOH, 100 MHz).

**Figure 5:**
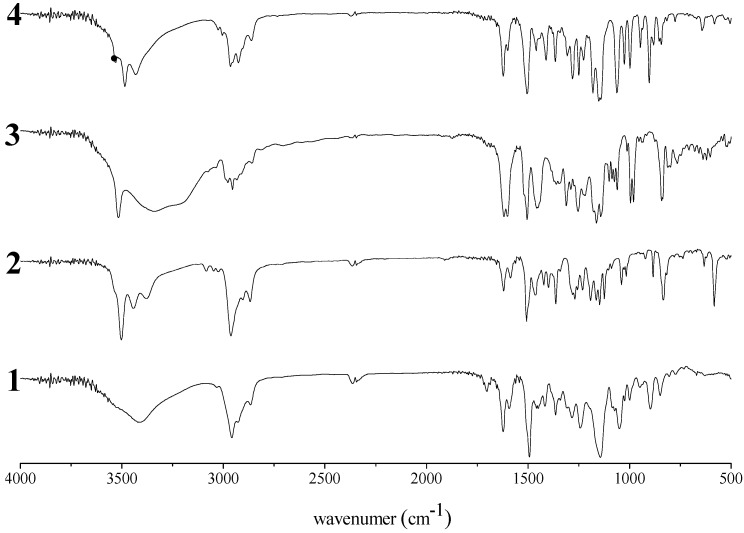
FT-IR spectra of **1**, **2**, **3,** and **4** (KBr).

The ^1^H-NMR, ^1^^3^C-NMR, and FT-IR spectra of polymer **1** are shown in Figure 2, Figure 3, Figure 4 and Figure 5, respectively. It was very difficult to determine the structure of the polymer from these spectra as the polymerization of resorciniol with acetone was complex. In a previous paper, it was reported that a chromane ring was formed after the the reaction of resorcinol with acetone in dilute solution [10]. On the basis of a previous study, we assumed that polymerization proceeded *via* simultaneous addition-condensation and cyclization of resorcinol with acetone. Therefore, the obtained polymer **1** was composed of the three types of repeating units (Unit A–C) as shown in Scheme 1. 

### MALDI-TOF mass study

**Figure 6:**
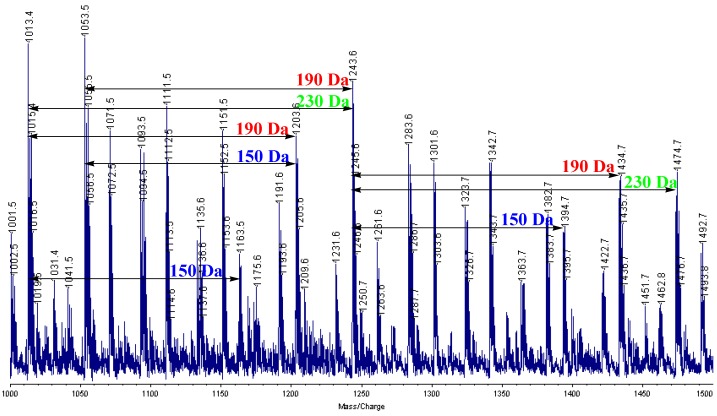
MALDI-TOF mass spectrum of polymer **1**.

In order to confirm the existence of the three types of repeating units, we measured the MALDI-TOF mass spectrum (Figure 6). Fragmentation patterns of polymer **1** showed the presence of regular peaks, which were formed according to the expression [m/z] = 150 Da (derived from **A**), 190 Da (derived from **B**), and 230 Da (derived from **C**). Therefore, this result suggested that polymer **1** was composed three different types repeating units (**Units A–C**).

### Model compounds and assignments

**Figure 7:**
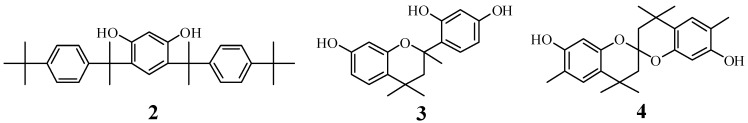
Model compounds **2**, **3**, and **4**.

In order to confirm the structure of the obtained polymer, we synthesized three model compounds, namely, **2** (isopropylidene bridge), **3** (chromane ring), and **4** (spiro-shaped double chromane ring) (Figure 7). Their structures were confirmed by the FT-IR, ^1^H-NMR, and ^13^C-NMR spectra. By comparing the model compounds **2-4** with polymer **1**, we confirmed the existence of the three repeating units as follows.

### Unit A: isopropylidene bridge

Because the ^1^H-NMR and ^13^C-NMR peaks of **1** were almost identical to those of **2**, it was confirmed that **1** contained an isopropylidene bridge structure. The observation of the upfield shift of the ^13^C-NMR quaternary carbon (Ph-C(CH_3_)_2_-Ph) peak in **1** (30–31 ppm), as compared to that in **2** (40.5 ppm) showed that hydroxyl group were not attached to both the terminal benzene rings (Figure 3 and Figure 4).

### Unit B: chromane ring; Unit C: spiro-shaped double chromane ring

From the NMR analysis, we also confirmed the existence of the chromane and spiro-shaped double chromane rings in polymer **1**. The ^1^H-NMR peaks of **1** were in good agreement with those of **3** and **4** (Figure 2). In particular, in the ^13^C-NMR spectra (Figure 3 and Figure 4), three peaks at 44 (R-CH_2_-R), 77 (R-CH_2_-C(CH_3_)(OPh)-R), and 97 (R-CH_2_-C(OPh)_2_-CH_2_-R) ppm were found to be completely consistent with those of **3** and **4**, suggesting that **1** possessed chromane and spiro ring structures. It was difficult to distinguish between **Unit B** and **Unit C** by analyzing the spectroscopic data.

### Content of phenolic hydroxyl group

**Scheme 2:**
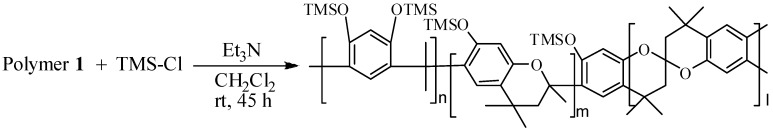
Synthesis of TMS (trimethylsilyl group)-modified polymer.

It was difficult to determine the ratio of each unit in the polymer by analyzing the spectroscopic data. However, the phenolic hydroxyl group content of the polymer can be determined by etherification. We therefore prepared the corresponding trimethylsilyl (TMS) derivatives of polymer **1** according to Scheme 2. From the FT-IR spectrum of the obtained polymer, it was observed that the peak of the phenolic hydroxyl group completely disappeared; therefore, the polymer reaction had proceeded quantitatively. From the ^1^H-NMR analysis, the integral ratio of the aromatic protons and the trimethylsilyl group protons was found to be ca. 2:5. This result suggested that for every repeating unit in polymer **1** there were 0.6 eq of hydroxyl groups. The numbers of hydroxyl groups contained in **Unit A**, **Unit B**, and **Unit C** were 2, 1, and, 0, respectively, therefore, it can be assumed that a large number of **Unit B**s (chromane ring) and **Unit C**s (double chromane ring) exist in the polymer. The ratios of *l*, *m*, and, *n* of each repeating unit could not be determined in the present study.

### High molecular weight polymer

In order to improve the processability of the obtained polymer, we tried to prepare a high molecular weight polymer by acid-catalyzed addition-condensation of polymer **1** and formaldehyde. However, the abovementioned reaction yielded a cross-linked insoluble gel. This is because the 2-position of the resorcinol skeleton showed high reactivity for addition-condensation; therefore, a cross-linking reaction efficiently proceeded under this reaction condition (see Experimental). Hence, to obtain a high molecular weight polymer, we selected a 2-substituted resorcinol, i.e., 2-methylresorcinol as the starting material. Polymer **5** (*M*_w_ = 2600, *M*_w_/*M*_n_ = 1.7) was prepared by the same procedure as that used to prepare polymer **1** using 2-methylresorcinol with acetone. Next, we successfully prepared an organosoluble high molecular weight polymer by the reaction of polymer **5** with formaldehyde. The obtained polymer was well soluble in THF and chloroform, and it exhibited a film-forming property. The *M*_w_ of the polymer was found to be 94,000 (*M*_w_/*M*_n_ = 4.3).

**Scheme 3:**
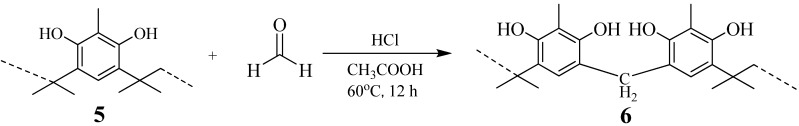
Synthesis of high molecular weight polymer **6**.

**Figure 8:**
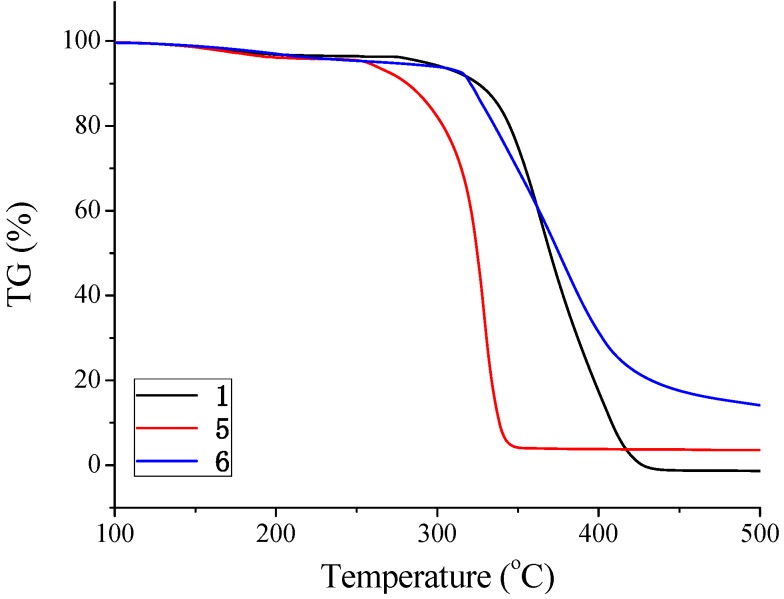
TG analysis of polymers **1**, **5**, and, **6**.

### Thermal properties

Differential scanning calorimetric (DSC) and thermogravimetric analyses (TGA) were carried out to determine the thermal transition and degradation behavior of the obtained polymers. From the DSC analysis, it was found that these polymers did not exhibit a glass transition temperature. From the TG analyses of **1**, **5**, and, **6**, the *T*_d10_ (10% loss in weight) was found to be 325, 281, and, 321 °C, and the polymers exhibited high thermal stability under nitrogen atmosphere. The spiro-shaped structure of the polymer backbone may be responsible for its thermal stability (Figure 8).

## Conclusions

In conclusion, we have synthesized an organosoluble resorcinol-acetone copolymer in the presence of acid catalyst. From the experimental results, especially the fragmentation patterns of the corresponding MALDI-TOF mass spectrum, and a previous report [11], we concluded that the obtained polymer consisted of the three types of repeating units, namely, isopropylidene-bridged resorcinols, chromane rings, and spiro-shaped double chromane rings. Even though the structure of the polymer was complex, it has a wide range of applications. The obtained polymer can be applicable not only for the development of plastic materials [11,12,13,14] such as thermosets, adhesives, and coatings but also for the synthesis of biomaterialss [15,16,17,18,19,20,21,22,23,24,25] such as antimicrobial agents, pesticides, and medicines. Applications for the epoxy resin and the evaluation of the antimicrobial ability are now under study.

## Experimental

### General

Unless otherwise noted, all reagents and chemicals were used without further purification. Paraformaldehyde (95%) and magnesium were obtained from Nacalai Tesque. 2-Methylresorcinol, 4-*tert*-butylbenzoic acid methyl ester, and trimethylsilyl chloride were obtained from TCI. Resorcinol and 4-methylresorcinol were obtained from Wako Pure Chem. and Aldrich, respectively. All the ^1^H-NMR spectra (in DMSO-*d*_6_, CDCl_3_-*d*_1_, or CD_3_OD-*d*_4_) and the ^13^C-NMR spectra (in DMSO-*d*_6_ or CD_3_OD-*d*_4_) were recorded on a 400 MHz JEOL LNM-EX400 instrument with tetramethylsilane (TMS) as the internal standard. The FT-IR spectra were recorded using a JASCO FT-IR 460 plus spectrometer. Gel permeation chromatography (GPC) was carried out by a JASCO UV-2070 detector and a JASCO RI-2031 detector (TOSOH TSKgel G3000H_XL_ or G4000H_XL_ column) using tetrahydrofuran (THF) as the eluent after calibration with polystyrene standards. Thermogravimetric analysis (TGA) was performed using a SII TG/DTA 6200 machine (SEIKO Instrument Inc.) with a heating rate of 10 °C/min under a nitrogen atmosphere. MALDI-TOFF mass was performed using Shimadzu Biotech Axima ToG 2.7.2. 

### Preparation of resorcinol-acetone copolymer **1** and 2-methylresorcinol-acetone copolymer **5**

Trifluoroacetic acid (98%, 10 mL) was added dropwise at 0 °C to a solution of resorcinol (5.5 g, 50 mmol) and an excess of acetone (15 mL). After stirring for 12 h at room temperature, the mixture was poured into water (300 mL) and the precipitate formed was collected. The obtained polymer was dissolved into minimum volume of acetone, and then the solution was reprecipitated in water (100 mL). The obtained polymer was dried *in vacuo* to give the polymer **1** (9.8 g). A similar polymerization of 2-metylresorcinol afforded a corresponding polymer **5** (12.5 g). **1**: *M*_n_ = 1,500, *M*_w_ = 3,700, *M*_w_/*M*_n_ = 2.3.; ^1^H-NMR (DMSO- *d*_6_, ppm): δ = 9.41-8.97 (-OH), 7.38-5.65 (Ph-H), 2.95-2.60, 2.12-0.31 (-CH_3_, -CH_2_-); ^13^C-NMR (DMSO-*d*_6_, ppm): δ = 156.2-148.0, 127.1-120.3, 109.2, 105.0-96.3, 77.6-76.8, 47.6-44.9, 34.0-32.0, 30.8-27.9; IR (KBr, cm^-1^): ν = 3,420, 2,960, 2,870, 1,622, 1,490, 1,240, 1,140, 1,050.; **5**: *M*_n_ = 1,500, *M*_w_ = 2,600, *M*_w_/*M*_n_ = 1.7.; ^1^H-NMR (DMSO- *d*_6_, ppm): 9.13-8.76 (-OH), 8.62-7.89, 7.25-5.89 (Ph-H), 3.01-2.63, 227-0.31 (-CH_3_, -CH_2_-); ^13^C-NMR (DMSO-*d*_6_, ppm): δ = 154.9-149.4, 129.5-128.6, 124.2-119.5, 113.3-105.6, 78.4-77.5, 47.2-44.5, 33.9-27.5, 10.2-8.30.; IR (KBr, cm^-1^): ν = 3,390, 2,960, 2,870, 1,610, 1,480, 1,430, 1,220, 1,080.

### Syntheses of Model Compound **2** (Scheme 4)

Dry diethyl ether (100 mL) and iodomethane 5 mL (79 mmol) was added dropwise to magnesium turnings (1.7 g, 70 mmol) in a flask at 0 °C. After stirring at room temperature for 0.5 h and then at 50 °C for 2 h, 4-*tert*-butylbenzoic acid methyl ester (3.8 mL, 20 mmol) was added at room temperature. The resulting mixture was stirring at room temperature for 0.5 h and then at 50 °C for 1 h. After cooling to room temperature, the resulting mixture was quenched with methanol (50 mL) and then 10% aqueous HCl (50 mL). The mixture was extracted with diethyl ether (3×100 mL) and the combined organic layer was dried over MgSO_4_. After removal of solvent, 2-(4-*tert*-butylphenyl)propan-2-ol was obtained (3.85 g, >99% yield). ^1^H-NMR (CDCl_3,_ ppm): δ = 7.43 (d, *J* = 8.8 Hz, 2H), 7.37 (d, *J* = 8.8 Hz, 2H), 1.56 (s, 6H), 1.32 (s, 9H); ^13^C-NMR (DMSO-*d*_6_, ppm): δ = 147.8, 147.4, 124.2, 124.1, 70.3, 33.8, 31.8, 31.1; IR (KBr, cm^-1^): ν = 3,340, 2,970, 2,860, 1,510, 1,460, 1,400, 1,360, 1,270, 1,170, 1,150, 1,130, 1,120, 960, 830.

**Figure 9:**
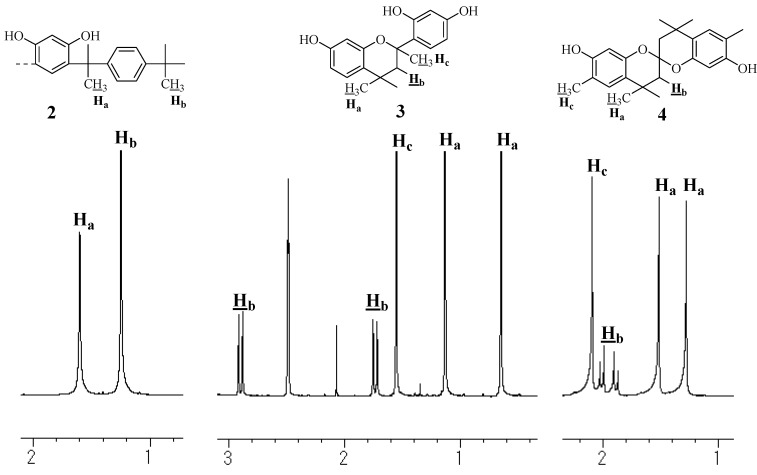
^1^H-NMR spectra of **2**, **3** (DMSO, 400 MHz), and **4** (MeOH, 400 MHz).

**Figure 10:**
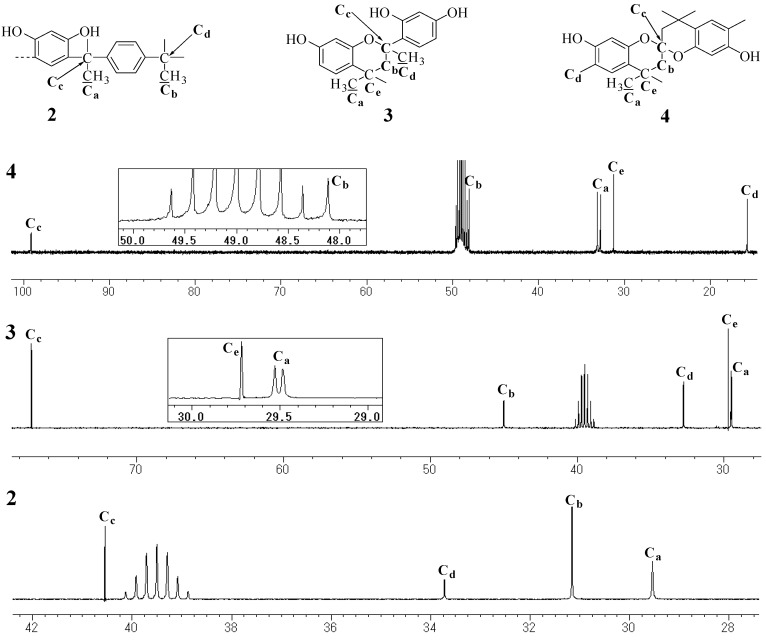
^13^C-NMR spectra of **2**, **3** (DMSO, 100 MHz), and **4** (MeOH, 100 MHz).

Next, hydrochloric acid (12 N, 0.1 mL) was added dropwise at 0 °C to a solution of resorcinol (0.11 g, 1 mmol), 2-(4-*tert*-butylphenyl)propan-2-ol (0.36 g, 2 mmol) and acetic acid (3 mL). After stirring 9 h at room temperature, the mixture was extracted with 3×50 mL of diethylether. The solution was concentrated under reduced pressure to afford the crude product. Futher purification was carried out by recrystallization from hexane to give the model compound **2** (0.27 g, 60% yield). ^1^H-NMR (DMSO-*d*_6, _ppm, Figure 9): δ = 8.44 (s, 2H), 7.21 (d, *J* = 8.4 Hz, 4H), 7.15 (s, 1H), 7.09 (d, *J* = 8.4 Hz, 4H), 6.09 (s, 1H), 1.60 (s, 12H), 1.25 (s, 18H); ^13^C-NMR (DMSO-*d*_6_, ppm, Figure 10): δ = 153.7, 148.4, 146.4, 125.2, 124.9, 124.3, 124.0, 104.5, 40.5, 33.7, 31.2, 29.5; IR (KBr, cm^-1^): ν = 3,500, 3,440, 3,380, 2,960, 2,870, 1,620, 1,510, 1,360, 1,270, 1,150, 1,120, 1,040, 830.

**Scheme 4:**
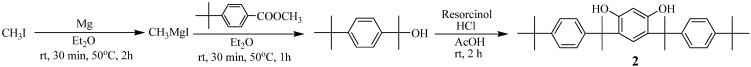
Syntheses of Model Compound **2**.

### Synthesis of model compound **3** (Scheme 5)

A mixture of resorcinol (6.6 g, 60 mmol), acetone (1.2 g, 20 mmol), hydrochloric acid (12 N, 2 mL), diethylether (100 mL), and CH_2_Cl_2_ (100 mL) was stirred and refluxed for 4 h. After removal of solvents, the yellow oil was poured into water. This obtained precipitate was dried in *vacuo* for 24 h to give the product **3** as colorless crystals (0.59 g, 20% yield). ^1^H-NMR (DMSO-*d*_6,_ ppm, Figure 9): δ = 6.93 (d, *J* = 8.1 Hz, 1H), 6.83 (d, *J* = 8.5 Hz, 1H), 6.27-6.23 (m, 3H), 6.03 (d, *J* = 2.4 Hz, 1H), 6.01 (d, *J* = 2.4 Hz, 1H), 2.90 (d, *J* = 13.9 Hz, 1H), 1.74 (d, *J* = 13.9 Hz, 1H), 1.56 (s, 3H), 1.14 (s, 3H), 0.65 (s, 3H); ^13^C-NMR (DMSO-*d*_6_, ppm, Figure 10): δ = 156.7, 156.1 , 154.5, 153.1, 127.0, 126.7, 121.9, 121.1, 108.2, 105.7, 103.2, 102.7, 77.1, 45.0, 32.7, 29.7, 29.5, 29.4; IR (KBr, cm^-1^): ν = 3,520, 3,340, 2,980, 2,960, 2,930, 1,620, 1,600, 1,510, 1,460, 1,310, 1,250, 1,160, 1,140, 1,000, 980, 840. 

**Scheme 5:**
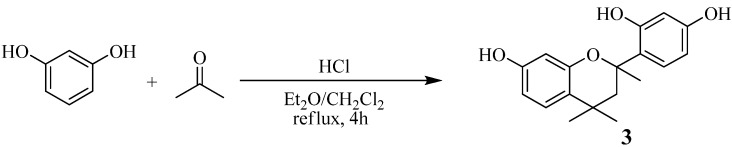
Synthesis of Model Compound **3**.

### Synthesis of model compound **4** (Scheme 6)

Acetone (64 μL, 1.5 mmol), acetic acid (0.2 mL), and hydrochloric acid (12 N, 0.2 mL) were added dropwise at 0 °C to a solution of 4-methylresorcinol (0.12 g, 1 mmol) and mercaptoacetic acid (46 μL, 1 mmol). After stirring 13.5 h at 80 °C, the mixture was extracted with diethyl ether (3×50 mL). The organic layer was dried over Mg_2_SO_4_. The resulting solution was concentrated under reduced pressure. The obtained residue was washed with an excess amount of acetone to afford the model compound **4** (0.07 g, 19% yield). ^1^H-NMR (CD_3_OD, ppm, Figure 9): δ = 6.9 (s, 2H), 6.00 (s, 2H), 2.10 (s, 6H), 2.01 (d, *J* = 13.9 Hz, 2H), 1.89 (d, *J* = 13.9 Hz, 2H), 1.52 (s, 6H), 1.28 (s, 6H); ^13^C-NMR (CD_3_OD, ppm, Figure 10): δ = 154.9, 150.4, 128.9, 123.6, 118.9, 104.3, 99.1, 48.1, 33.1, 32.8, 31.2, 15.7; IR (KBr, cm^-1^): ν = 3,530, 3,490, 3,430, 2,970, 2,930, 2,860, 1,620, 1,510, 1,410, 1,370, 1,280, 1,250, 1,180, 1,150, 1,060, 1,020, 1,000, 900.

**Scheme 6:**
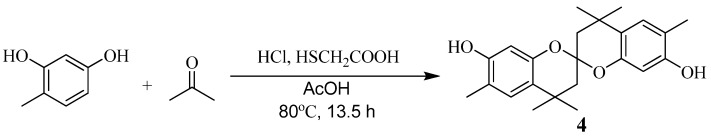
Synthesis of Model Compound **4**.

### Preparation of high molecular weight polymer **6**

2-Methylresorcinol-acetone copolymer **5** (0.98 g, 6.0 mmol) and paraformaldehyde (0.12 g, 3.0 mmol) were dissolved in acetic acid (8 mL) at room temperature. After stirring for a few minutes, the mixture temperature drop to 0 °C and then hydrochloric acid (12 N, 1 mL) was added dropwise. After stirring 12 h at 60 °C, the mixture was poured into water (100 mL) and the precipitate was collected. Further purification was carried out by HPLC. The high molecular weight polymer **6** was obtained as a colorless precipitate (0.97 g, 92% yield). *M*_n_ = 22,000, *M*_w_ = 94,000, *M*_w_/*M*_n_ = 4.3.; ^1^H-NMR (DMSO-*d*_6,_ ppm): δ = 8.60-7.99, 7.17-6.26 (Ph-H), 3.88-3.53 (Ph-CH_2_-Ph), 2.96-2.60, 2.28-0.36 (-CH_3_, -CH_2_-); IR (KBr, cm^-1^): ν = 3,400, 2,960, 2,930, 2,870, 1,610, 1,480, 1,440, 1,220, 1,080, 930.

### TMS-modified polymer

To a solution of **1** (0.15 g) and Et_3_N (0.77 mL, 5.5 mmol) in CH_2_Cl_2_ (5 mL) was added TMSCl (0.54 g, 5 mmol). After stirring at room temperature for 45 h, the mixture was extracted with dichloromethane (3×50 mL). The solution was concentrated under reduced pressure to give the crude product, which was dissolved in a minimum volume of acetone, and reprecipitated in methanol (50 mL). The obtained precipitate was dried *in vacuo* to give the TMS-modified polymer **1** (0.086 g). *M*_n_ = 1,300, *M*_w_ = 3,300, *M*_w_/*M*_n_ = 2.5.; ^1^H-NMR (CDCl_3,_ ppm): δ = 7.22-6.78, 6.62-5.81 (Ph-H), 2.82-2.62, 2.13-0.53 (-CH_3_, -CH_2_-), 0.40-0.095 (-SiCH_3_); IR (KBr, cm^-1^): ν = 3,390, 2,960, 2,870, 1,610, 1,480, 1,430, 1,220, 1,080.
